# Cemented or uncemented fixation: Which allows a more acceptable prosthetic femoral version in total hip arthroplasty?

**DOI:** 10.1186/s13018-023-04331-1

**Published:** 2023-12-09

**Authors:** Maria Moralidou, Anna Di Laura, Harry Hothi, Johann Henckel, Alister J. Hart

**Affiliations:** 1 Institute of Orthopaedics and Musculoskeletal Science, University College London, Brockley Hill, Stanmore, HA7 4LP UK; 2 The Royal National Orthopaedic Hospital NHS Trust, Brockley Hill, Stanmore, HA7 4LP UK; 3 Department of Mechanical Engineering, University College London, Torrington Place, London, WC1E 7JE UK; 4 Cleveland Clinic London Hospital, 33 Grosvenor Pl, London, SW1X 7HY UK

**Keywords:** Primary total hip arthroplasty, Prosthetic femoral version, Uncemented hip surgery, Cemented hip surgery

## Abstract

**Background:**

Three-dimensional computed-tomography (3D-CT) planning for primary Total Hip Arthroplasty (THA) typically uses the external femoral surface; as a result, it is difficult to predict the prosthetic femoral version (PFV) for uncemented femoral stems that press-fit to the internal surface of the bone. Cemented fixation allows the surgeon to adjust the version independent of the internal femoral anatomy. We aimed to better understand the effect of the fixation type on PFV.

**Methods:**

This was a case series study including a total of 95 consecutive patients (106 hips), who underwent uncemented (*n* = 81 hips) and cemented (*n* = 25 hips) primary THA using the posterior approach. The surgeon aimed for a PFV of 20°. Our primary objective was to compare PFV in both groups; our secondary objective was to evaluate the clinical outcomes.

**Results:**

The mean (± SD) PFV was 13° (± 9°) and 23° (± 8°) for the uncemented and cemented THA groups (*P* < 0.001), respectively. In the uncemented THA group, 36% of the patients had a PFV of < 10°. In the cemented THA group, this clinically important threshold dropped to 8%. Similarly, the Bland–Altman (BA) plots showed wider 95% limits of agreement for the uncemented group. Satisfactory clinical outcomes were recorded.

**Conclusion:**

We found that the PFV was more clinically acceptable, for the posterior surgical approach, in the cemented group when compared to the uncemented group. Both THA groups reported high variability indicating the need to develop surgical tools to guide the PFV closer to the surgical target.

## Background

Previous computed-tomography (CT) studies have reported a high variability of prosthetic femoral version (PFV) in primary total hip arthroplasty (THA), ranging from − 17° to 72° [1–13]. The accuracy in measuring version angles using Two-Dimensional (2D) cross-sectional CT images is lower when compared to Three-Dimensional (3D)-CT model-based measurements [14–16]. Existing literature has highlighted a lower discrepancy between 3D-CT and dry bone measurements than using single 2D cross-sectional scans, concluding that the 3D-CT method is the virtual equivalent of the reference standard (dry bone measurements) [14]. Studies using 3D-CT analysis have highlighted an increased incidence of prosthetic femoral retroversion and a wide range of PFV (− 23° to 43°) [16–26] (Table 1).

**Table 1:** CT-measured PFV in previous studies

Ref	N	CT/3DCT	Uncem. /Cem	Stem Design	PFV (Mean ± SD, Range) [Deg]	RAS/Nav	SA	CO
Suh [2]	33	CT	Uncem	Straight Non-Anatomic Versus Fibre Metal Taper (Zimmer)	18 ± 6 (3–28)	No	P	NA
Wines [3]	111	CT	Uncem./Cem	29 C. Ted (Smith and Nephew)/75 C. Less (Synergy)/7 Other Design	17 ± 11 (− 15 to 45)	No	80 P/31 L	0% Dis
Reikeras [4]	91	CT	Uncem	Straight Stem (Corail, Depuy)	23 ± 12 (− 17 to 60)	No	40 L/51 P	0% Dis., 0% Rev
Nakashima [5]	111	CT	Uncem	Straight Metaphyseal Fit-Fill (Kyocera)	34 ± 11 (9–60)	No	P	0% Dis
Hirata [6]	73	CT	Uncem	Straight Metaphyseal Fit-Fill (Kyocera)	35 ± 11 (9–60)	No	P	0% Dis
Fujishiro [7]	1411	CT	Uncem	Straight Stem	40 ± 11 (0–72)	No	P	Na
Hirata [8]	122	CT	Uncem	Straight Metaphyseal Fit-Fill (Kyocera)	38 ± 11 (14–63)	No	P	0% Dis
Fujishiro [9]	1555	CT	NA	NA	40 ± 12	No	P	3.2% Dis
Okada [10]	81	CT	Uncem	Taper Wedge (Accolade II, Stryker)	27 ± 5 (17–39)	Yes	AL	0% Dis
Jackson [11]	29	CT	Uncem	Straight Stem (Corail, Depuy)	22 ± 11	Yes	A	NA
Hochreiter [12]	12	CT	Uncem	6 Calcar-Guided Short Stems /6 Straight Stems	Calcar-Guided: 23 ± 5.5/Conv.:14 ± 7	No	AL	NA
Imai [13]	65	CT	Uncem	Straight Metaphyseal Fit-Fill (Kyocera)	32 ± 10 (12–58)	Yes	L	0% Dis
Dorr [17]	47	3DCT	Uncem	Anatomic Porous (APR; Zimmer)	11 ± 8 (− 9 to 27)	Yes	P	NA
Sariali [18]	223	3DCT	Uncem	SPS-Modular (Symbios)	27 ± 14	No	183 AL/ 40P	NA
Sendtner [19]	60	3DCT	Uncem	Straight Stem (Corail, Depuy)	6 ± 11 (− 19 to 33)	No	A	NA
Kiernan [20]	60	3DCT	Cem	ScanHip System (Biomet)	20 (1–43)	No	P	1% Rev
Inoue [21]	65	3DCT	Uncem	Short Fit-Fill Anatomical Stem -Aps Natural-Hip System (Zimmer)	19 ± 9 (− 2 to 39)	No	P	NA
Park [16]	19	3DCT	Uncem	Collarless Tapered Wedge Stem (Linear Stem; DJO Global)	19 ± 9 (0–34)	Yes	P	NA
Dimitriou [22]	19	3DCT	Uncem	Collarless Tapered Wedge Stem (Linear Stem; DJO Global)	11 ± 13 (− 23 to 33)	No	P	NA
Weber [23]	123	3DCT	Uncem	Straight Stem (Corail, Depuy)	8 ± 10 (− 19 to 38)	No	AL	NA
Hayashi [24]	44	3DCT	Uncem	Tri-Lock Bps Stem (Depuy)	31 ± 10	No	AL	NA
Nodzo [25]	20	3DCT	Uncem	Restoris Femoral Stem (Stryker)	9 ± 6	Yes	P	0% Dis
Belzunce [26]	30	3DCT	Uncem	Straight-Tapered (Quadra-H, Medacta)	14 ± 10 (− 5 to 39)	No	P	3% Dis

*Cem.* Cemented, *Uncem.* Uncemented, *RAS* robotic-assisted surgery, *Nav* navigation, *SA* surgical approach, *CO* clinical outcome, *NA* not available/not applicable, *A* anterior, *P* posterior, *L* lateral, *AL* anterolateral, *Dis* dislocation, *Rev* revision

The PFV of an uncemented femoral stem is partly dictated by the stem design and the highly variable internal morphology of the proximal femur [27]. Consequently, the final stem position is a compromise of best-fitting a straight stem down to the proximal femur, leaving the surgeon with minimal control over the PFV [23, 28, 29]. Contrastingly, in cemented femoral stems, the surgeon can intra-operatively adjust the version of the femoral stem to a desired position within the variable thickness of the cement mantle [17, 30, 31].

Suboptimal placement of the femoral stem with regards to its version is associated with the biomechanical instability of the reconstructed hip joint [20, 32, 33]. Impingement has been reported common in uncemented femoral stems with a PFV of < 5° [30], and a low PFV is associated with increased dislocation rate via the posterior approach [9, 27]. Furthermore, a revision rate of 40% has been reported among stems with a PFV of < 10° [20].

Considering the lower revision and dislocation rates that have been reported in the cemented femoral stems when compared to the uncemented femoral stems [34–36] and the relationship between the PFV and potential adverse clinical effects, we aimed to better understand the effect of the fixation type on PFV. Our primary objective was to measure the PFV in uncemented and cemented THA. Our secondary objective was to measure clinical outcomes. Our hypothesis was that cemented fixation, using a collarless double-tapered femoral stem, offers greater control of PFV than uncemented straight femoral stems.

## Materials and methods

### Study design

This was a retrospective study including 95 patients (106 hips) who underwent primary THA between February 2017 and June 2021 due to osteoarthritis. We divided the patients into two groups based on the stem fixation technique adopted, uncemented and cemented (Table 2). The surgery was performed through a posterior approach by a single consultant orthopaedic surgeon who specialises in hip arthroplasty.

**Table 2:** Study group characteristics

	Uncemented group (*n* = 81 hips)	Cemented group (*n* = 25 hips)	*P* value
Gender (females) (%)	40 (49)	14 (56)	0.57
Age (years) (median, range)	62 (32–86)	64 (42–89)	0.45
Treatment side (right) (%)	42 (52)	17 (68)	0.16
NFV (deg) (median, range)	14° (7–20°)	14° (10–18°)	0.87

A single hemispheric press-fit Hydroxyapatite (HA) coated cup was used in both groups, two stem designs were used, straight-tapered in the uncemented and collarless double-tapered in the cemented group.

The outcome measures were as follows:

1. PFV angles.
2. Cup version angles
3. Clinical outcomes.

### Pre-operative radiology and 3D software planning

All patients underwent pre-operative CT scanning of the hip region and the knee joint according to a standard protocol. A PS femoral neck osteotomy guide was designed, using the pre-operative CT data. In addition, 3D pre-operative planning was performed to establish the optimal acetabular and femoral implant size and position (MyHip, Medacta International SA, Castel San Pietro, Switzerland). During the operation, the surgeon aimed for a PFV of 20°.

3D-CT models of the pre-operative femurs were generated to measure NFV using Simpleware ScanIP software (Version 2021.03; Synopsys, Inc., Mountain View, USA) [16].

### Surgical approach

All surgeries were performed through a posterior approach by a single consultant orthopaedic surgeon.

### Prosthetic components

In the uncemented THA group, a straight-tapered femoral stem was used (Quadra-H; Medacta International SA, Castel San Pietro, Switzerland); in the cemented THA group, a collarless double-tapered femoral stem (X-Acta system; Medacta International SA, Castel San Pietro, Switzerland) was used. A hemispheric press-fit Hydroxyapatite (HA) coated cup was used for both groups (Mpact system; Medacta International SA, Castel San Pietro, Switzerland).

### Patient-specific instrumentation (PSI)

During the surgery, a PS femoral neck osteotomy guide was used to perform the osteotomy. The guide was 3D-printed to fit the contours of the femoral head-neck junction. During the surgery, the sterilised PS cutting jig was positioned on the femoral head-neck junction and two pins secured its position. The surgeon then performed the osteotomy with the oscillating saw blade flush on the surface of the guide. The femoral neck osteotomy plane was defined as a plane inclined by 45° to the long axis of the proximal femur.

### Post-operative radiology

All patients underwent post-operative CT scanning of the hip region and the knee joint that was done according to a standard protocol. Post-operative evaluation took place; number of fractures and dislocations was recorded. Oxford hip score (OHS) of cases reporting complications was recorded. 3D models of the post-operative femurs and prosthetic components were generated, using Simpleware ScanIP software (Version 2021.03; Synopsys, Inc., Mountain View, USA). PFV angles were measured, to assess the impact of the fixation method on the PFV.

### Prosthetic femoral version (PFV) and acetabular cup version

**Fig. 1:**
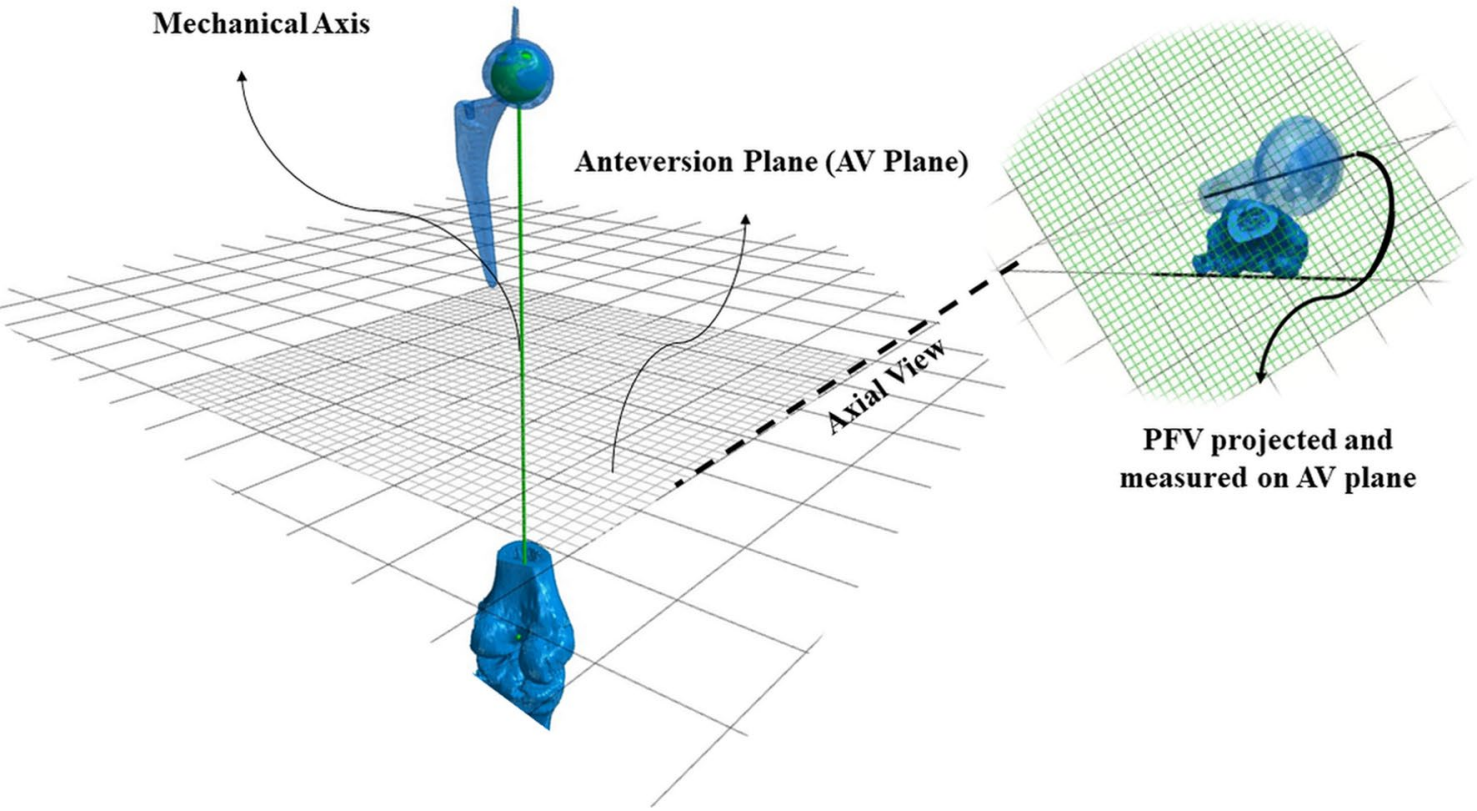
Illustration of the coordinate system used to measure the PFV

Post-operative PFV was measured, as the angle between the axis of the neck of the femoral stem and the posterior condylar axis (PCA) projected on a plane perpendicular to the mechanical axis of the reconstructed femur (femoral stem) (Fig. 1) [19]. The stem neck axis was defined as the line connecting the centre of the head with the top mark of the stem [26].

Post-operative acetabular cup version was measured in the radiographic definition using the anterior pelvic plane (APP) [37, 38]. The cup plane was computed as the best-fitted plane based on 10 points chosen on the cup rim.

### Reproducibility and reliability analysis

We measured the reproducibility of our CT measurement method using intra and interobserver analysis. For the intraobserver analysis, the same user measured PFV twice for 30 randomly selected cases, while for the interobserver analysis, a second user ran the test twice for 20 randomly selected cases.

Measurements of PFV were also obtained using an independent commercially available software (ZedHip, LEXI Co, Ltd, Tokyo, Japan).

### Statistical analysis

SPSS software was used to perform the statistical analysis (version 28, SPSS, Chicago, USA). The Shapiro–Wilk test was used to evaluate the normality of the data in both groups and Mann–Whitney *U* test was implemented to evaluate differences between the two groups with regard to the study group characteristics. The median and interquartile range (IQR) were estimated for NFV angles. The mean, median, IQR and standard deviation (SD) were estimated for PFV angles. We compared the NFV and PFV for each case using a Bland–Altman (BA) plot.

The data describing the PFV angles were of different sample size and variance. Therefore, we performed the Welch’s test to assess if the mean values of PFV in both THA groups were statistically different.

For the reproducibility and reliability analysis, mean and SD of differences between the measurements of the same and different users were reported. Intraclass correlation coefficient (ICC) was obtained for both intra- and interobserver reliability.

## Results

### Discrepancy between NFV and PFV of individual cases in both THA groups

**Fig. 2:**
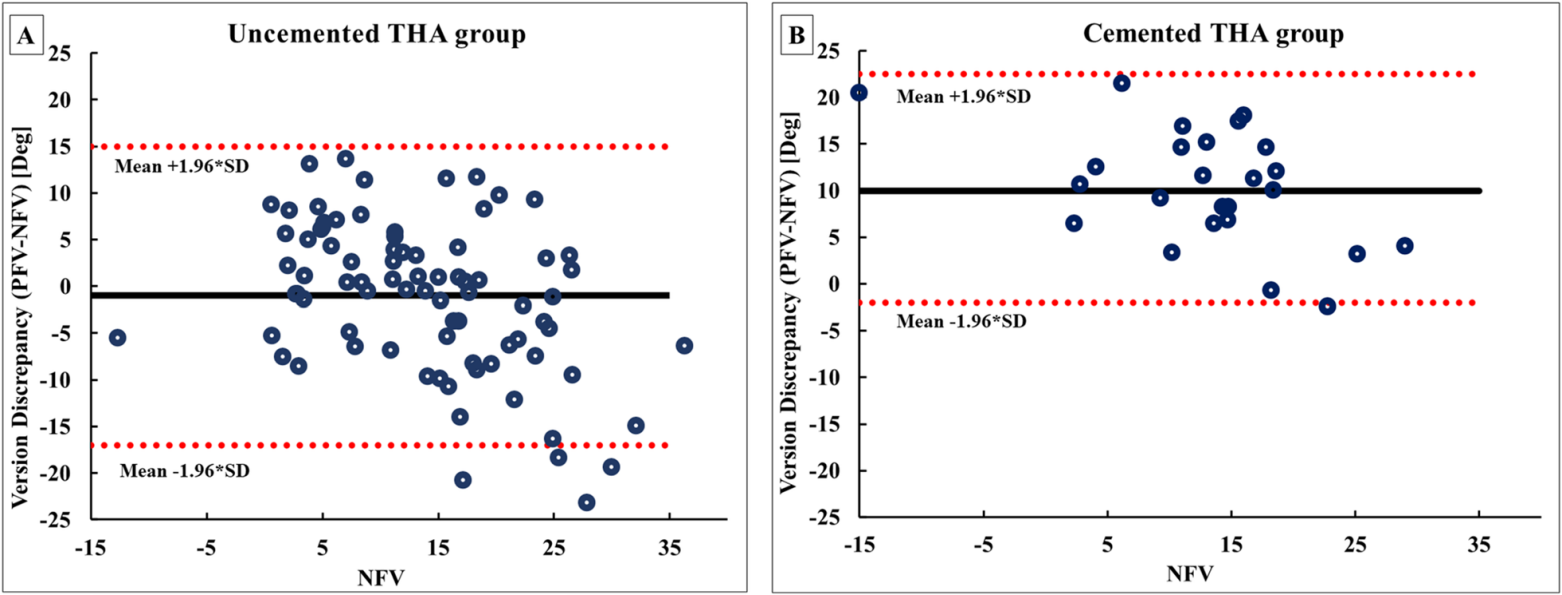
BA plot of the comparison between NFV and PFV of individual cases in (**A**) the uncemented THA group and (**B**) the cemented THA group

A BA plot of the discrepancy between NFV and PFV showed a mean discrepancy of − 1° and 10° of version for the uncemented and cemented groups, respectively. Furthermore, the uncemented THA cases had 95% limits of agreement between − 17 and 15°. This was wider than the cemented THA cases, where the 95% limits of agreement were − 2 and 22.5° (Fig. 2).

### PFV in uncemented and cemented THA

**Fig. 3:**
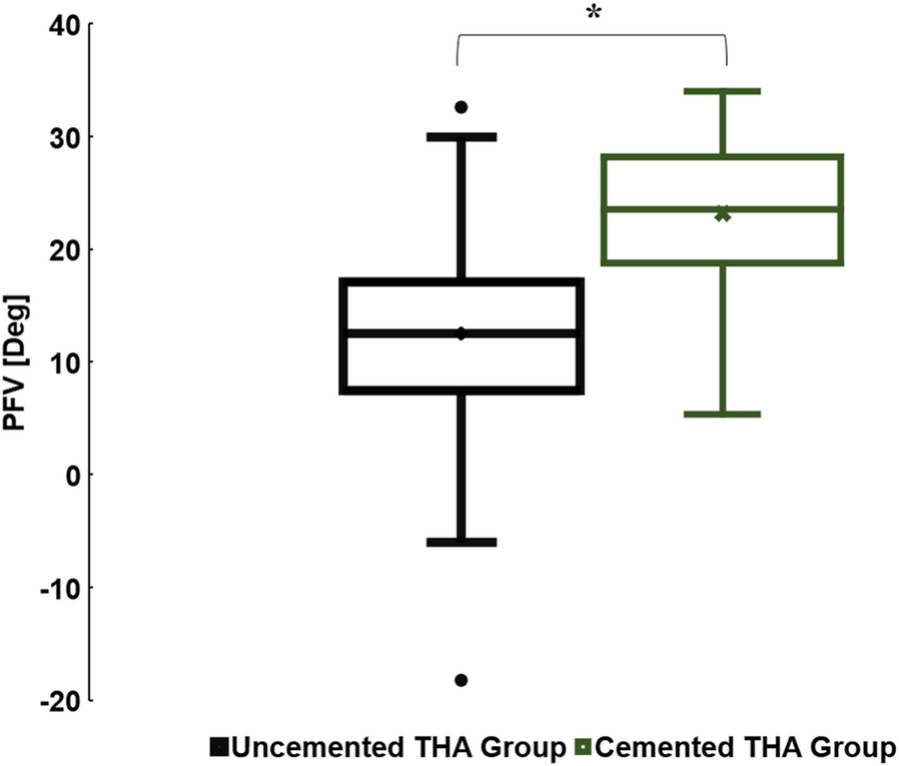
Box plot illustrating the PFV in the uncemented and cemented THA groups (*P < 0.001)

The uncemented THA had a mean (± SD) and median (IQR) PFV of 13° (± 9°) and 13° (8–17°), respectively. The cemented THA group had mean (± SD) and median (IQR) PFV of 23° (± 8°) and 24° (18–28°), respectively. We found a statistically significant difference between the mean values of PFV in the uncemented and cemented THA groups (*P* < 0.001) (Fig. 3).

In the uncemented THA group, PFV measurements ranged from -18° retroversion to 33° anteversion. Five patients in the uncemented THA group had retroverted PFV. Four of these patients had a PFV of − 5° ± 1° and one had a PFV of − 18°. The NFV of these patients was 17°, 1°, 3°, 2° and − 13°, respectively. In addition, the absolute difference between the PFV and NFV was 21°, 6°, 9°, 8° and 5°, respectively.

In the cemented THA group, PFV values were ranged between 5° and 34°. There were no retroverted femoral stems in this group.

**Fig. 4:**
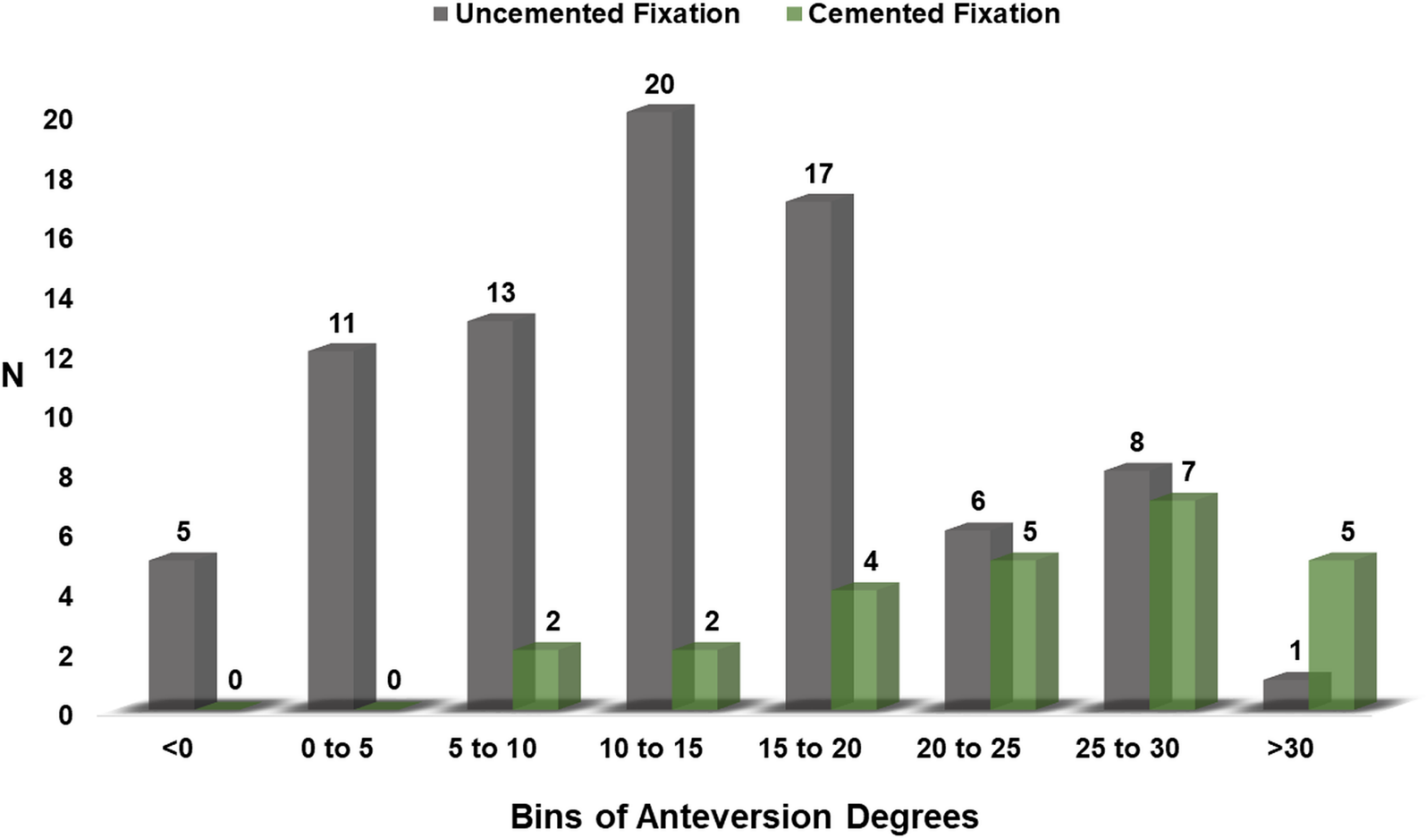
Distribution of PFV in uncemented and cemented THA groups

In the uncemented THA group, a PFV of between 5° and 10° and between 10° and 15° was reported in 16% and 25% of the femoral stems, respectively. Twenty-one per cent (21%) of the uncemented femoral stems had a PFV of between 15° and 20° and 7% had a PFV of between 20° and 25°. Finally, 11% of the uncemented femoral stems were anteverted of between 25° and 35° and 20% were anteverted of less than 5° (Fig. 4).

Concerning the distribution of PFV in the cemented THA group, 8% of the femoral stems reported a PFV between 5° and 10° and between 10° and 15°. A PFV of between 15° and 20° and between 20° and 25° was reported in 16% and 20% of the cemented femoral stems, respectively. Forty-eight per cent (48%) of the cemented femoral stems were anteverted more than 25° (Fig. 4).

### Acetabular cup version in uncemented and cemented THA

The uncemented THA had a mean (± SD) and median (IQR) acetabular cup version of 23° (± 8°) and 23° (17–28°), respectively. The cemented THA group had mean (± SD) and median (IQR) acetabular cup version of 26° (± 7°) and 25° (20–30°), respectively.

### Reproducibility and reliability analysis

We achieved excellent intraobserver repeatability and interobserver reproducibility in PFV. In any case, the ICC was more than 0.99, while the mean (± SD) difference between the same and different raters was 0.01 ± 1° and − 0.4 ± 2°, respectively.

The mean (± SD) difference of PFV measured by the external software (ZedHip, LEXI Co, Ltd, Tokyo, Japan) and our method was − 1 ± 2°.

### Clinical outcomes

The median follow-up time was 45 and 21 months for the uncemented and cemented series, respectively. Satisfactory clinical outcome was recorded without any intra-operative fracture and post-operative implant loosening. Two dislocations were reported in the uncemented THA group: one as a result of deep hip flexion and one during yoga (child’s pose), 5 weeks and 12 months after the surgical procedure, respectively. Treatment included one closed reduction procedure with no further surgeries up to 48 months post-operatively. There was no recurrence of the dislocation and the OHS was 48/48 for both cases at 1 year post-operatively. The NFV for the two dislocated cases was 18° and 8°, respectively. Post-surgery, both cases reported a PFV of 9°, while the absolute difference between the PFV and NFV for these cases were 9° and 1°, respectively. In addition, the acetabular cup version was 29° and 8°, while the combined version (CV) was 38° and 17°, respectively.

## Discussion

This was the first study to assess the impact of the fixation method on the PFV (the achieved version of the femoral stem) using 3D-CT image analysis. We found that PFV in the cemented THA group was higher when compared to the uncemented THA group (*P* < 0.001). In the uncemented THA group, 36% of the patients reported a PFV of < 10°, with 5 patients having a retroverted PFV. This percentage dropped to 8% in the cemented THA group. There were not any retroverted femoral stems in the cemented THA group.

Table 1 includes all the studies assessing the PFV using CT scans or 3D-CT analysis. High variability of PFV has been reported, ranging from − 23° to 72°. In this study, the PFV of an uncemented straight-tapered femoral stem ranged between − 18° and 33°.

Previous literature addressing the effect of the fixation method on PFV using Magnetic Resonance Imaging (MRI) scans reported a lower PFV in cemented femoral stems when compared to the findings of the present study (13 ± 8° vs. 23 ± 8° in our study) [39]. They declared 2.3% of retroverted cemented femoral stems and 11.8% of retroverted uncemented femoral stems. In our study, there were no retroverted femoral stems in the cemented THA group, but there were 6% of retroverted uncemented femoral stems. The difference may be explained by the different designs of the femoral stems used.

The findings of this study are consistent with the increased surgeon control of the position of cemented femoral stem designs and highlight that by intra-operatively adjusting the PFV, using the malleable nature of the cement mantle, the surgeons can avoid delivering a retroverted or insufficient PFV. This information is clinically relevant, considering the importance of an adequate PFV to eliminate undesirable events like dislocation or impingement in primary THA [9, 30, 36].

It is true that not only cemented femoral stems can offer intra-operative control of the femoral stem position. Uncemented femoral stems featuring modular necks allow modularity of the femoral stem neck in various configurations of PFV [18]. These components, however, have been linked to a risk of mechanical failure [40]. In contrast, the excellent implant survivorship reported for the cemented femoral stem designs [41] indicates that the cemented fixation is a safe alternative to deliver an adequate PFV in primary THA.

For straight, uncemented femoral stems, the femoral implant is tightly press-fitted into the bone to achieve the so-called best-fit position within the medullary canal of the proximal femur, leaving the surgeon with minimal control over the PFV [29]. For this reason, Dorr et al. [17] have emphasised the importance of delivering a CV (the sum of the acetabular and femoral version angles) between 25° and 50° to avoid dislocation in primary uncemented THA. In this context, when pre-operative planning indicates the risk of a suboptimal PFV due to either an excessive or retroverted patient’s NFV, surgeons can consider adjusting the cup version to compensate for an abnormal PFV, using the approach of the femur first technique [42].

However, despite its importance for avoiding dislocation [5, 17], the concept of a CV within the optimal range does not guarantee an optimal version for the individual prosthetic components (e.g., a case with a PFV of − 10° and a cup version of 40°). Recent studies have highlighted that focusing solely on the cup version to define a universal safe optimum for hip motion is not sufficient [43], and even when the generally accepted optimal range of CV is achieved, dislocations are not infrequent [44].

Current commercial planning solutions cannot predict the PFV of a straight uncemented femoral stem. Despite the high accuracy of 3D pre-operative planning in predicting implant size [45, 46], with the potential to minimise implant inventory [47], the surgical plan does not always deliver the targeted PFV in uncemented THA. PFV demonstrates an increased variability, ranging from − 19° of retroversion to 33° of anteversion [19]. This information implies the need to develop novel designs of intra-operative measuring tools that could potentially measure the version of the femoral broach. Surgeons could then classify the cases where uncemented fixation could not deliver an adequate PFV and choose cemented femoral stem designs instead.

Nevertheless, the greater adjustability that a cemented femoral stem may offer did not seem to lower the SD of the PFV in the cemented THA group. Intra-operative adjustment of PFV using the cement mantle is subjected to the surgeon’s perspective. Intra-operatively guiding the PFV of a cemented femoral stem, using either robotic tools or 3D-printed customised surgical guides, may be considered beneficial.

Particular emphasis should be given to femoral broaching and the implantation of a cemented femoral stem. Differences in the PFV of the femoral broach and of the implanted cemented femoral stem would result in an asymmetrical thickness of the cement column. Existing literature has supported that the thickness of the cement mantle around the femoral stem impacts cement strains [48], stem subsidence [49], micro-movement at the cement–bone interface [50], and the overall long-term radiographic outcome [51]. The presence of defects may affect the fixation interfaces and potentially function as an area of osteolysis [52–54]. However, there is still a debate about the optimal cement thickness [55, 56]. Long-term follow-up clinical studies are needed to determine if an asymmetrical cement mantle thickness negatively affects the implant’s survival.

Furthermore, considering that there is a continuing debate around the most appropriate fixation technique in primary THA, the findings of this study suggest that PFV may constitute an additional criterion during the selection process. A limited number of studies so far, have reported higher dislocation, revision and loosening rates in the uncemented primary THA, when compared to the cemented THA [34–36]. In this context, it is probable that this increased prevalence of untoward events in uncemented THA may stem from the high variability of PFV [17]. Long-term clinical studies are imperative to identify any significant association between PFV and post-operative clinical outcome in uncemented and cemented THA.

We acknowledge limitations. Firstly, PFV angles were measured based on 3D-CT reconstructed models of the proximal femurs and prosthetic models using a standardised coordinate system; this procedure is considered as a virtual equivalent to the reference standard [16]. The main limitation of this method is the amount of subjectivity induced by landmarks selection. Excellent intra and interobserver analysis proved that the PFV measurements were not significantly influenced by the user input. For the measurement of NFV measurements we used a published method [16]. Our measurements of NFV values are in accordance with those of previous studies [16, 29, 57].

Secondly, the findings may depend on the geometry of the femoral stems used, not fully reflecting the influence of cemented femoral stems of a different geometry on PFV. We compared a fit-fill, uncemented femoral stem of a trapezoidal cross-section with a narrower, highly polished, cemented femoral stem. A potential comparison between uncemented and cemented femoral stems of similar geometry would have resulted in less difference in PFV and the incidence of retroversion.

Lastly, the two THA groups had unequal sample sizes and follow-up time. Considering the fact that dislocation has been reported to occur within the first 12 months of the surgery [58, 59], we assumed that the follow-up time of the cemented THA group (21 months) is considered an adequate follow-up time to detect any clinically adverse effects.

## Conclusions

Recent CT studies have reported a high variability of PFV in uncemented THA, suggesting that the internal morphology of the proximal femur may affect the final version of the femoral component. In this study, we found that the use of a cemented fixation technique led to higher PFV, when compared to the uncemented group although both groups reported a similar variability. With cemented fixation, surgeons have greater control of PFV. There is need to develop surgical tools that can intra-operatively measure and/or guide version of the femoral component.

## Data Availability

The data is available from the corresponding author on reasonable request.

## Abbreviations

APP: Anterior pelvic plane
BA: Bland–Altman
CT: Computed-tomography
CV: Combined version
HA: Hydroxyapatite
ICC: Intraclass correlation coefficient
IQR: Interquartile range
NFV: Native femoral version
OHS: Oxford hip score
PCA: Posterior condylar axis
PFV: Prosthetic femoral version
PS: Patient-specific
PSI: Patient-specific instrumentation
SD: Standard deviation
3D-CT: Three-dimensional computed-tomography
THA: Total hip arthroplasty
